# Excretion of *Eimeria* spp. oocysts in young lambs following iron supplementation

**DOI:** 10.1186/s13028-018-0404-6

**Published:** 2018-08-29

**Authors:** Ane Odden, Synnøve Vatn, Antonio Ruiz, Lucy Jane Robertson, Heidi Larsen Enemark, Silje Katrine Nes, Vibeke Tømmerberg, Snorre Stuen

**Affiliations:** 1 Department of Production Animal Clinical Sciences, Faculty of Veterinary Medicine, Norwegian University of Life Sciences, Kyrkjevegen 332/334, 4325 Sandnes, Norway; 2 Animalia Norwegian Meat and Poultry Research Centre, P.O. Box 396 Økern, 0513 Oslo, Norway; 3 Department of Animal Pathology, Faculty of Veterinary Medicine, University of Las Palmas de Gran Canaria, Arucas, 35416 Las Palmas, Spain; 4 Department of Food Safety and Infection Biology, Faculty of Veterinary Medicine, Norwegian University of Life Sciences, P.O. Box 8146 Dep, 0033 Oslo, Norway; 5 Department of Animal Health and Food Safety, Norwegian Veterinary Institute, Ullevålsveien 68, P.O. Box 750 Sentrum, 0106 Oslo, Norway

**Keywords:** Coccidiosis, *Eimeria* spp., Iron supplementation, Norway, Sheep

## Abstract

**Background:**

Iron is an essential nutrient, and iron supplementation has been shown to reduce the incidence of abomasal bloat in lambs. Additionally, iron deficiency is linked to pica, which may increase uptake of *Eimeria* oocysts. Coccidiosis in sheep, caused by *Eimeria* spp., is an important infection, leading to reduced welfare and economic losses. The aims of our study were to investigate: (1) the use of iron supplementation in Norwegian sheep flocks using a questionnaire survey, and (2) whether iron supplementation reduced excretion of *Eimeria* oocysts and increased the growth rates of young lambs.

**Results:**

A questionnaire regarding the use of iron supplementation, sent to all members of the Norwegian Sheep Recording System (n = 4993), showed that 152/1823 farmers iron-supplemented lambs, either orally (56.7%) or by injection (43.3%). The main purpose of supplementation was to prevent abomasal bloat (38.4%), coccidiosis (9.3%), or both (27.8%). In the field study, 102 twin lambs from five flocks were included: one twin (treated) received 600 mg of gleptoferron subcutaneously within 3 days of birth, whereas the control was given saline. McMaster analysis of individual faecal samples obtained at weekly intervals (n = 4 per lamb, starting at turnout) showed no significant difference in oocyst excretion between treatment groups at any sampling, except for one flock 14 days after turnout. Mean growth rates, measured at iron injection, 21 days after turnout, and in the autumn, differed significantly between treated and untreated lambs from iron injection to 21 days after turnout, however, no difference in growth rates was observed in the overall period from iron injection to autumn. Blood analysis suggested that the controls were at risk of developing iron deficiency anaemia during the housed period, but signs of anaemia were not observed.

**Conclusion:**

Iron supplementation of lambs was used by 8.3% of the farmers responding to the questionnaire, mainly with the intention to prevent abomasal bloat, coccidiosis, or both. The field trial results indicate that iron supplementation of young lambs do not reduce oocyst excretion and only induced a transitory increase in weight gain. However further studies, including more flocks and possibly repeated iron injections, would provide more definitive information.

**Electronic supplementary material:**

The online version of this article (10.1186/s13028-018-0404-6) contains supplementary material, which is available to authorized users.

## Background

Iron is an essential element in all living organisms, including as an important component or cofactor in many proteins and enzymes, such as haemoglobin and myoglobin [1]. Due to rapid growth, low iron content in milk, and no access to soil, which is the main source of dietary iron for farm animals [2–4], housed lambs may develop anaemia. Iron deficiency anaemia is well recognised, both in housed piglets [5–7] and in housed lambs [8–12]. Dietary deficiency in iron may lead to pica, i.e. ingestion of material other than normal food, including soil [13]. In Norway, anaemia is occasionally seen in connection with abnormal appetite and development of abomasal bloat in lambs [11, 14]. Pica in lambs on spring pasture, leading to ingestion of excessive amounts of soil, could potentially result in uptake of high numbers of *Eimeria* spp. oocysts as they can survive for at least 1 year in soil under Norwegian conditions [15].

In Norway, most ewes are winter housed, and lambing occurs in March–May, followed by turnout to spring pastures 1–4 weeks post-partum [16, 17]. During summer, ewes and lambs normally graze on mountain, forest or otherwise uncultivated pastures, before the lambs are weaned in the autumn, at around 4–5 months of age [16]. Lambs become infected with *Eimeria* spp. either during the housed period or immediately after turnout [15]. Coccidiosis in sheep caused by *Eimeria* spp. leads to reduced welfare, increased mortality, and substantial production losses [18–20]. Clinical signs of coccidiosis include abdominal pain, anorexia, diarrhoea (± haemorrhagic) and weight loss/reduced growth [21]. Control strategies include adequate nutrition, hygienic measures, and pasture rotation [22, 23]. However, prevention of outbreaks in Norway is largely based on chemoprophylaxis with anticoccidials, usually with toltrazuril treatment at turnout or about 1 week later [24, 25]. Resistance in poultry has been reported for several anticoccidials [26, 27]. In addition, toltrazuril resistance has been confirmed in a field isolate of *Cystoisospora suis* [28]. Widespread use of anticoccidials in Norway, combined with unverified reports of reduced anticoccidial efficacy in ovine *Eimeria* spp. [25, 29], accentuate the importance of alternative control strategies.

Previous research has indicated that iron supplementation of lambs might increase growth rates and prevent abomasal bloat [12, 14]. These results have prompted the current guidelines for iron supplementation in Norwegian sheep flocks, which recommend the use of iron supplementation for prevention of abomasal bloat [30]. The aims of our study were therefore: (1) to map the use of iron supplementation in Norwegian sheep flocks based on a questionnaire survey, and (2) to investigate whether iron supplementation of young lambs reduces the uptake and excretion of *Eimeria* oocysts and increases lamb growth rates, thus, potentially, reducing the need for treatment with anticoccidials.

## Methods

### Questionnaire

A questionnaire on iron supplementation in lambs was sent by email to all members of the Norwegian Sheep Recording System (NSRS) with a registered email address, using the Enalyzer Survey Solution (Enalyzer A/S). Membership in the NSRS is voluntary, and 36.5% of all farmers were members in 2016, representing 47.9% of all ewes in Norway and all sheep producing counties [2]. A translated copy of the questionnaire can be found as Additional file 1. Farmers (n = 4993) who received the questionnaire, represented 32.2% of all sheep flocks in Norway [31]. Non-responding farmers were reminded once.

### Iron supplementation trial

The study on investigation of the effect of iron supplementation of young lambs on *Eimeria* oocyst excretion was approved by the Norwegian Animal Research Authority, ID 8535. The CONSORT statement was used as a guideline in the design of the study [32].

Five flocks (A–E) located in Rogaland County, in Southwest Norway, were included in the study, which was performed during April and May, 2017. Flocks were selected based on known clinical problems with coccidiosis (unpublished data), and proximity to the laboratory at Norwegian University of Life Sciences (NMBU), Sandnes, Norway. Twin pairs born within a period of 6 days were selected from each flock. The twins were randomly allocated (coin toss) to either iron supplementation (treated) or control groups. Treated lambs were injected with 600 mg gleptoferron (Gleptosil vet., Ceva Santé Animale, France) subcutaneously in the inguinal fold, 0–3 days after birth. At the same time, the twin was injected with a corresponding volume (3 mL) of 9 mg/mL sterile NaCl (B. Braun Melsungen AG, Germany). Lambs were housed with their dam for 16–31 days before turnout, which was considered day 0. All included lambs were kept on slatted floors (plastic in flocks A, B, and E, and expanded metal in flocks C and F). In flock C, 18 lambs (9 treated and 9 controls) were kept for about 1 week on solid floors with wood shavings after injection. All five flocks used cultivated pastures for spring grazing, and all pastures had been grazed by lambs during the previous year. The farmers treated against helminths at around 3 weeks after turnout using either benzimidazole or ivermectin.

**Fig. 1:**
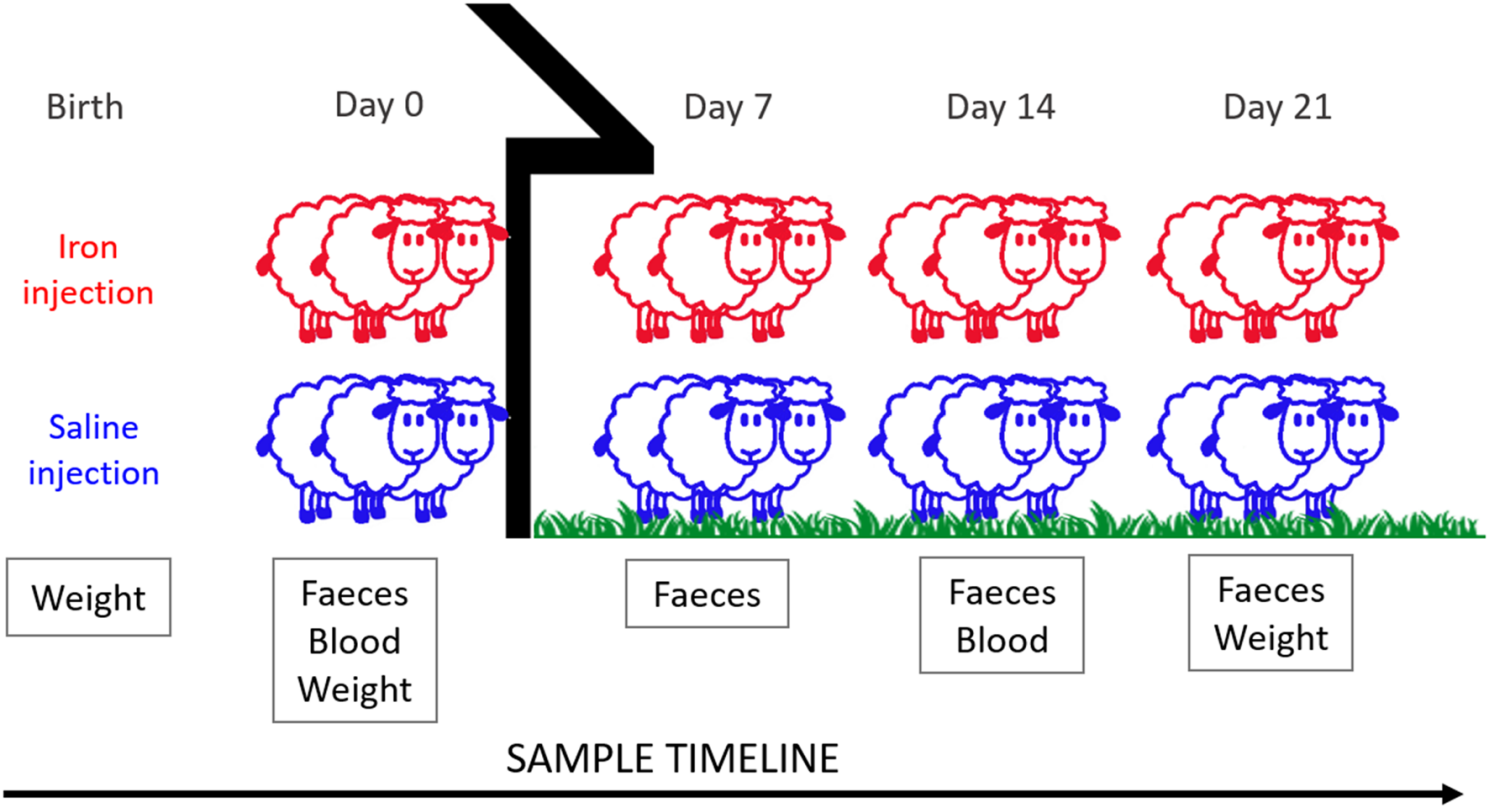
Sample protocol. Twin lambs (n = 20) in five flocks, injected with iron or saline 0–3 days after birth, were sampled according to the given protocol. Days are given as days after turnout

Faecal samples were taken at day 0 (turnout), 7, 14 and 21 (Fig. 1). All samples were collected individually in zip-lock bags and vacuum packed (Fresh ‘n’ easy, OBH Nordica, Sweden) on the day of sampling, and stored at 4 °C until analysis within 28 days. Faecal samples were analysed using a modified McMaster technique with a minimum theoretical sensitivity of 5 oocysts per gram (OPG) [33, 34]. *Eimeria* were not identified to the species level. Additionally, the faecal consistency was scored visually on a scale from one to five: (1) normal, pelleted; (2) soft; (3) liquid; (4) watery; (5) watery with blood and/or intestinal tissue [35]. Scores ≥ 3 were regarded as diarrhoeic.

Weights were recorded at iron injection (day − 16 to − 31), day 21, and day 79–150. Blood were drawn from a jugular vein using a vacutainer system (plain and EDTA-treated, BD Company, USA) at day 0 and 14. Haematology was performed immediately using the ADVIA 120 Haematology system (Bayer Diagnostics, Germany). The main haematological parameters evaluated were red blood cell counts (RBC), haemoglobin (HGB), and haematocrit (HCT). Whole blood tubes were centrifuged within 2 h, and serum was stored at − 20 °C. Serum iron (Fe) was analysed by ABX Pentra 400 (Horiba, France). Internal reference limits (NMBU, Sandnes) for blood parameters were calculated based on previous results [36, 37].

Toltrazuril was administered to two lambs from flock D, both iron treated, at day 21 due to severe diarrhoea. No other lambs were treated with anticoccidials.

### Statistical methods

Data were managed in Excel 2013 (Microsoft Inc., USA). Statistical analyses were performed in Stata 14 (Stata Statistical Software: Release 14. StataCorp LP, College Station, TX, USA), and graphs were made in R [38]. T-tests were used for calculations of significance based on means, except for oocyst counts for which Mann–Whitney U-tests were used, due to lack of normality. Fisher’s exact tests were used to evaluate correlations. P< 0.05 was considered significant.

## Results

### Questionnaire

The dataset from the questionnaire consisted of 1822 complete and 36 incomplete answers, corresponding to a response rate of 38.1%. When possible, data from the incomplete questionnaires were included in the analysis, and thus *n* varies between calculations. Iron supplementation in lambs were used by 152 of 1823 farmers (8.3%). Farmers using iron supplementation were mainly located in Oppland (40.1%), Rogaland (15.8%), or Hedmark (9.9%) counties. The mean flock size was 95.5 ± 1.9 winter-fed ewes (range 3–800), with a significant difference (P < 0.01) between non-supplementing (90.7 ± 1.8, range 3–610) and supplementing (148.9 ± 9.8, range 29–800) flocks. Table 1 shows the administration route and the purpose of the treatment.

**Table 1:** Questionnaire data from Norwegian sheep farmers supplementing with iron

	%	n
Administration route		
Oral	56.7	85
Injection	43.3	65
Total		150
Purpose		
Abomasal bloat	38.4	58
Abomasal bloat and coccidiosis	27.8	42
Coccidiosis	9.3	14
Other/uncertain^a^	24.5	37
Total		151
Intend to supplement next year		
Yes	93.4	142
No	6.6	10
Total		152

^a^Other purpose/uncertain includes recommendations by veterinarian, experience of pica in lambs, and focus on increasing growth rates. n = number of farms

### Iron supplementation trial

In total, 102 lambs were included in the trial (22 lambs from flock A and 20 lambs from each of the flocks B–E). Age at turnout of the lambs ranged from 16 to 31 days (Table 2). In flock B, one lamb from the control group died 17 days after turnout, and post mortem revealed pneumonia associated with *Mannheimia haemolytica*. In flock E, two lambs were treated for pneumonia, one around turnout and another 14 days after turnout. These lambs were excluded from evaluation of growth rates and faecal analysis. In flock D, one lamb from the treated group died of unknown reasons on summer pasture.

**Table 2:** Twin lambs from five flocks (A–E) located in Rogaland County, Norway, included in an iron injection field trial

Flock	Number of lambs	Treated		Control		Age at iron injection (days)	Age at turnout (days)	Breed^a^
		Rams	Ewes	Rams	Ewes			
A	22	3	8	9	2	1–3	16–18	NWS
B	20	5	5	6	4	0–2	20–23	NWS
C	20	5	5	5	5	1–3	29–31	NWS and NST
D	20	4	6	4	6	0–3	16–21	NWS
E	20	4	6	5	5	2–3	16–17	NWS and NST

Lambs were either supplemented with 600 mg gleptoferron (treated) or physiological saline (controls) subcutaneously

^a^*NWS* Norwegian White Sheep, *NST* Norwegian Short Tail

There was a significant difference in mean growth rates (g/day) between treated and untreated lambs in the period from iron injection to 21 days post turnout, where treated lambs had higher mean growth rates than controls. However, at the flock level, this difference was only found in flock E (Table 3). There were no differences in mean growth rates from day 21 after turnout to autumn or from iron injection to autumn (day 79–150). The two lambs from flock D treated with toltrazuril at day 21 was not removed from calculation of growth in the autumn, as their growth rate was not significantly different from the other iron treated lambs.

**Table 3:** Mean growth rates (g/day, mean ± SEM) of iron supplemented lambs and controls in the five flocks (A–E)

	Iron injection^a^—21 days after turnout		21 days after turnout—autumn^b^		Iron injection—autumn^b^	
	Treated	Control	Treated	Control	Treated	Control
A	392 ± 19	357 ± 19	177 ± 12	203 ± 14	224 ± 12	238 ± 14
B	394 ± 14	371 ± 12	294 ± 17	324 ± 37	324 ± 14	334 ± 22
C	374 ± 11	332 ± 25	248 ± 28	251 ± 25	287 ± 19	276 ± 16
D	410 ± 13	423 ± 22	252 ± 12	249 ± 10	293 ± 12	295 ± 13
E	367 ± 17*	311 ± 15	345 ± 27	367 ± 26	351 ± 21	351 ± 20
All flocks	388 ± 7*	359 ± 10	262 ± 12	274 ± 13	295 ± 9	296 ± 9

Treated lambs were subcutaneously supplemented with iron within 3 days of birth. Turnout was considered day 0

* P < 0.05

^a^Iron injection: days − 31 to − 16

^b^Autumn: days 79 to 150

**Fig. 2:**
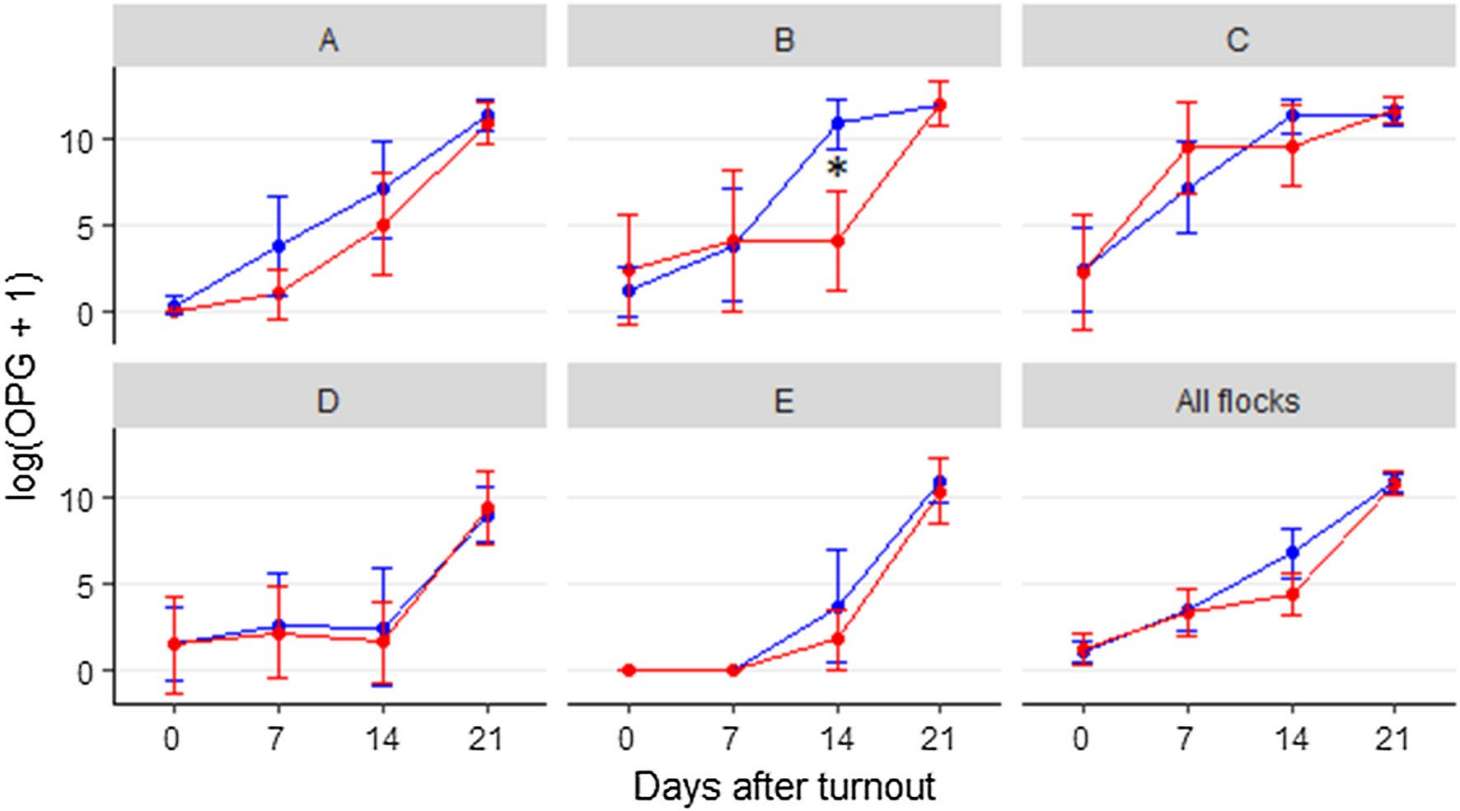
Mean oocyst excretion in 102 twin lambs supplemented subcutaneously with iron (red) or saline (blue). Lambs from five Norwegian sheep flocks (A–E) with known coccidiosis problems were sampled at day 0, 7, 14 and 21 after turnout. *P< 0.05

Four of the five flocks were infected with *Eimeria* spp. during the housed period, i.e. oocysts were detected at turnout (day 0) (Fig. 2), and lambs in all five flocks excreted *Eimeria* oocysts (range 10–1,043,000 OPG) 14 days after turnout. Although OPG counts were lower in treated lambs than in untreated lambs at day 14 in all flocks, this difference was not statistically significant in any of the flocks, except flock B. In addition, there was no statistical significant difference in OPG between the treated and control lambs in any of the flocks at the other sampling dates. Maximum oocyst excretion for both groups of lambs and in all five flocks was observed at day 14 or 21.

Diarrhoea was observed during the study period. At turnout, one treated lamb from flock D had diarrhoea, whereas two lambs (one treated and one control) from flock C had diarrhoea on day 14. On day 21, the mean faecal score was < 2, except for in the control group in flock A, where the mean faecal score was 2.1 ± 0.3 (mean ± SEM). However, there was no significant difference in the faecal scores between treated and control lambs in any of the flocks at any sampling time.

Two flocks were positive for *Nematodirus battus* at day 21; in flock B, 77.8% of the lambs were positive (range 20–310 EPG) and in flock D, 25.0% were positive (range 10–50 EPG). However, presence of diarrhoea was not associated with detection of *N. battus.* Diarrhoea was only seen in two of the lambs diagnosed with *N. battus* in flock B, but in none of the *N. battus*-positive lambs in flock D. No other helminths were detected.

**Fig. 3:**
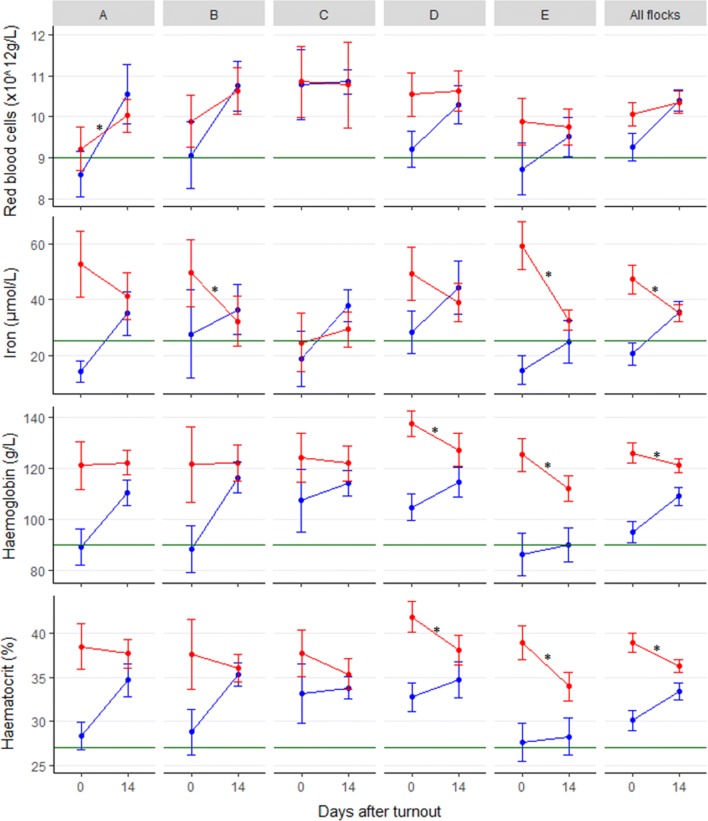
Mean blood levels of red blood cells, iron, haemoglobin and haematocrit with 95% confidence intervals for twin lambs in the five included flocks (A–E) at day 0 and 14 after turnout. Half of the lambs were supplemented with iron 16–31 days before turnout. Red: iron supplemented lambs, blue: control lambs, green line: lower reference limit (internal references). *Significant difference in the treated group between samplings (P< 0.05)

Except for in flock C, there was a significant difference in blood iron content between treated and control lambs at day 0 (Fig. 3), and the mean blood iron values in the control groups of flocks A, C, and E were below the reference limit of 25.0 µmol/L (internal references, NMBU, Sandnes). However, at day 14 after turnout, there was no difference in mean blood iron concentrations between treated and control groups in any of the flocks. In addition, a significant reduction in blood iron was seen in the treated group from turnout to day 14 in flocks B, E, and the whole dataset. A similar significant reduction between the treated groups’ samples was seen between day 0 and day 14 for HGB in flocks D, E, and the whole dataset, and for HCT in flocks D, E, and the whole dataset.

## Discussion

According to the questionnaire, iron supplementation was performed in 8.3% of the sheep flocks, amongst which more than 90% of the farmers intended to continue this practice. Moreover, more than 30% of the farmers that supplemented lambs with iron did so with the intention of preventing coccidiosis. An important finding from the questionnaire was the significant difference in flock size between flocks receiving iron supplementation and flocks that did not, with larger flocks more likely to practice iron supplementation than smaller flocks. The reason for this is unknown, but might reflect a shifted focus in the sheep industry from treatment of individual animals to a more preventive, flock health approach. Especially as the average flock size has increased in Norway over the last decade [31].

Few studies have investigated the effect of iron treatment of lambs on the excretion of *Eimeria* spp. and development of clinical coccidiosis. However, unpublished data (Vatn, personal communication) indicated a significant reduction in *Eimeria* oocyst excretion three to 5 weeks after turnout that was associated with iron supplementation. These findings were not supported by the present study, in which iron supplementation of lambs did not reduce excretion of *Eimeria* oocysts 3 weeks after turnout. This may indicate that reduction of geophagia by iron supplementation is not an efficient way to reduce *Eimeria* oocyst uptake and excretion in lambs. Regardless of the reason for decreased oocyst excretion in the study by Vatn, a similar reduction in oocyst excretion did not occur in our study despite the larger number of animals and farms included. There was however, an apparent reduction in oocyst excretion in iron supplemented lambs 2 weeks after turnout. Although this was mostly non-significant, the potential that this may reflect a delay in uptake and excretion of *Eimeria* oocysts might suggest that development of immunity could be affected. However, whether this occurred and whether this could confer some protection on the lambs is unknown.

Clinical signs of coccidiosis in lambs in Norway tend to occur 2–3 weeks after turnout, and it has been assumed that the lambs are primarily infected following ingestion of oocysts on permanent spring pastures [15, 39, 40]. Nevertheless, the present study shows that indoor infection with *Eimeria* spp. may not be unusual in Norway, as oocysts were detected in the faecal samples at turnout in four of the five flocks.

All five flocks participating in the treatment trial experienced diarrhoea and perianal soiling, signs related to both coccidiosis and nematodirosis [21, 41]. However, based on the parasitological analyses, the diarrhoea was not correlated with nematodirosis. Other gastrointestinal pathogens, such as rotavirus, coronavirus, *Cryptosporidium* spp., and *Salmonella* sp. are not commonly diagnosed in lambs in Norway and were not investigated in our study, and we cannot rule out that they may have had a role in the observed clinical signs.

Previous studies investigating effects of iron supplementation of lambs have used various dosages and iron preparations: e.g., Bassett et al. [8] administered 200 mg iron dextran intramuscularly within 24 h of birth, Vatn and Torsteinbø [14] injected 300 mg iron dextran subcutaneously to lambs within 1 week of birth, and in our study we used 600 mg gleptoferron subcutaneously within the first 3 days of life. In addition, Pollmann et al. [42] showed that there was no difference in serum Fe concentrations, serum Fe-binding capacities, RBC, HGB or HCT between piglets supplemented with iron dextran, compared to piglets supplemented with gleptoferron. The dose employed might be of importance, as the need for iron is largely dependent on growth rates; i.e., rapidly growing animals require iron to maintain haematopoiesis during the 1st weeks of life [9, 43]. The dose used in our study, 600 mg gleptoferron, should be sufficient to cover the lambs’ requirements. However, treated lambs showed significantly lower levels of iron, HGB and HCT at day 14, compared with their blood samples from day 0, indicating that their iron storage was low, and that higher or repeated doses of iron might have been beneficial. Should an increased or repeated iron dose be used, then the risk of reaching toxic levels must be evaluated. Clinical signs of acute iron toxicity in ruminants include anorexia, respiratory distress, icterus and central nervous signs [1, 44]. In our study, no signs related to iron toxicity were observed.

Iron supplementation of lambs may have a variable effect on lamb growth rates [8, 11, 14, 45–47]. In the present study, weight gain was not significantly affected by iron supplementation in any of the flocks when considering the growth period from birth to autumn. The difference in growth rates in the period from iron injection to 21 days post turnout (lamb age: 37–52 days) might be the result of iron supplementation, as control lambs in many cases showed blood values for iron below the reference level at turnout. However, although no significant differences were found, the control lambs grew better than the iron-supplemented lambs in four out of five flocks during the subsequent summer grazing period. This might be explained by the lambs’ capacity for compensatory growth [48]. In addition, it is important to remember that lamb growth is dependent on several other factors, such as nutrition [49], mastitis in the dam [50], and gastrointestinal helminths [51].

Blood values from the field trial lambs suggest that without iron supplementation, the lambs were at risk of developing anaemia, although none of the flocks showed associated clinical signs [52]. The significant difference in blood iron content between treated and untreated lambs at turnout, was largely absent 14 days later, indicating that the control lambs ingested iron and started producing red blood cells. In one of the flocks (C), differences in blood parameters between treated and untreated lambs could not be demonstrated. The lambs from this flock were around 1.5 weeks older than lambs from the other flocks, and might have started ingesting solid feed, such as concentrates, prior to turnout. Additionally, these lambs had access to wood shavings during the indoor period, which may also have affected the blood parameters.

The farmers reporting use of iron supplementation in the questionnaire were mainly located in the inland, mountainous areas (Oppland and Hedmark counties), whereas the field trial was performed in the Southwest coastal area (Rogaland county). This geographical difference might have affected our findings, as significant climatic variations between the regions are known [53]. Likewise, differences in the iron concentration of feed crops may vary between areas [54]. However, this is unlikely to have had a significant effect on the iron levels of the young lambs in this study.

## Conclusion

Iron supplementation was used by less than 10% of the sheep farmers responding to the questionnaire, and the purpose of treatment was mainly to prevent abomasal bloat, but also coccidiosis. However, in the field study, iron supplementation did not affect excretion of *Eimeria* oocysts by lambs, nor was it associated with increased growth rates. These results indicate that iron supplementation of young lambs does not provide an appropriate alternative control strategy for prevention of coccidiosis. However, further studies are needed in order to verify this statement by including more flocks, preferably from different geographical regions, and using higher or repeated doses of iron.

## Additional file

**Additional file 1.** A translated copy of the questionnaire sent to all members of the Norwegian Sheep Recording System.
