# Supplementary material for: Excretion of Eimeria spp. oocysts in young lambs following iron supplementation
Source: Acta Vet Scand. 2018 Aug 29;60:49. doi: 10.1186/s13028-018-0404-6 (PMC6114706; doi:10.1186/s13028-018-0404-6)
Supplement: Supplementary file 1 — Additional file 1. A translated copy of the questionnaire sent to all members of the Norwegian Sheep Recording System. [file 13028_2018_404_MOESM1_ESM.pdf]

Additional file

A translated copy of the questionnaire sent to members of the Norwegian sheep recording system (n=4993).

1. In which county is your farm located?
2. How many winter fed ewes do you have?
3. Did you supplement lambs with iron (injection/oral) in 2017?
    - Yes
    - No

If yes:

4. How was iron administered?
    - Orally
    - Injected
5. What was the purpose of the treatment?
    - Against abomasal bloat
    - Against coccidiosis
    - Against both abomasal bloat and coccidiosis
    - Other reason, please describe
6. Do you think supplementation had the effect you wanted?
    - Yes
    - No
7. Will you supplement lambs next year?
    - Yes
    - No
8. How old were the majority of your lambs at turnout?
    - 0-7 days
    - 8-14 days
    - 15-21 days
    - 22-28 days
    - 29-35 days
    - 36-42 days
    - > 42 days
9. Any additional comments regarding iron supplementation of lambs?
